# Total uterine prolapse: a rare cause of chronic obstructive uropathy associated with renal dysfunction (a case report)

**DOI:** 10.11604/pamj.2023.44.57.38550

**Published:** 2023-01-30

**Authors:** Anna Thanasa, Efthymia Thanasa, Ioannis Paraoulakis, Evangelos Kamaretsos, Apostolos Ziogas, Gerasimos Kontogeorgis, Vasiliki Grapsidi, Evangelos-Ektoras Gerokostas, Elisavet Mylona, Ioannis Thanasas

**Affiliations:** 1 Department of Health Sciences, Medical School, Aristotle University of Thessaloniki, Thessaloniki, Greece,; 2 Department of Obstetrics and Gynecology of General Hospital in Trikala, Trikala, Greece,; 3 Department of Obstetrics and Gynecology, University of Thessaly, Larissa, Greece,; 4 Department of Radiology of General Hospital in Trikala, Trikala, Greece

**Keywords:** Total uterine prolapse, hydronephrosis, renal dysfunction, imaging examination, case report

## Abstract

Pelvic organ prolapse is rarely associated with severe bilateral ureteral hydronephrosis and renal dysfunction. The etiopathogenetic mechanism has not been fully elucidated. Contemporary imaging methods of the urinary tract play a decisive role in assessing the morphological function of the kidneys. In cases of moderate and severe pelvic organ prolapse, surgery appears to be the main choice of treatment. Our case concerns a post-menopausal patient with three vaginal deliveries in her obstetric history and with a history of bilateral hydronephrosis and impaired renal function who was referred to the outpatient clinic for a gynecological examination due to complete uterine prolapse. Bilateral hydroureteronephrosis due to prolapse was assessed as the main cause of renal dysfunction. A surgical intervention was decided to the pelvic floor and a vaginal hysterectomy was performed with simultaneous correction of the cystocele and rectocele. The postoperative course was uneventful. Three months later, re-examination of the urinary tract showed complete remediation of kidney morphology and function. The present case report emphasizes the significant degree of bilateral hydroureteronephrosis and deterioration of renal function rarely seen in patients with complete uterine prolapse. At the same time, it is pointed out that the exclusion of renal dysfunction related to complete uterine prolapse should be the main concern of the modern gynecologist even for complex cases with coexisting etiological factors for renal disease, in order to avoid permanent renal parenchymal damage and ensure the best health and quality of life of these patients.

## Introduction

Uterine prolapse is an anatomical abnormality of the female reproductive system characterized by the descent, sliding or downward displacement of the uterus and/or cervix and their adjacent organs, such as the bladder and/or rectum, due to the weakening of the supporting structures of the pelvic floor [1]. Depending on its severity, uterine prolapse is divided into four stages: from stage I, in which the most distant part of the prolapse is more than one centimeter above the level of the hymen, to stage IV (our case), which is characterized by complete vaginal eversion [2]. Uterine prolapse may cause obstructive uropathy with unilateral or bilateral mild hydronephrosis [3]. The prevalence of hydronephrosis from pelvic organ prolapse is not easy to determine with accuracy, mainly due to the heterogeneity of the various statistics, which is probably due to the small number of patients with prolapsed uterus undergoing imaging of the urinary system. In general, it is estimated to vary between 3.5% and 30.6%, regardless of the stage of pelvic organ prolapse [4,5]. In this article, after the description of the case, a brief literature review of complete uterine prolapse-related bilateral hydroureteronephrosis, accompanied by a severe deterioration of renal function, is attempted.

## Patient and observation

**Patient information:** a post-menopausal 59-year-old patient with a known history of complete uterine prolapse under conservative management with vaginal pessary placement was referred from the nephrology clinic to the gynecology outpatient clinic to undergo a gynecological examination. The patient had three vaginal deliveries in her obstetric history and medical history of Hashimoto's thyroiditis, diabetes mellitus type 2, and hyperlipidemia, which were kept under control with medication treatment. She was hospitalized a few days ago in the pathology clinic of our hospital, due to a urinary tract infection and severe deterioration of renal function. From the medical history, neither chronic renal disease was reported, nor recurrent urinary tract infections in recent years.

**Figure 1:**
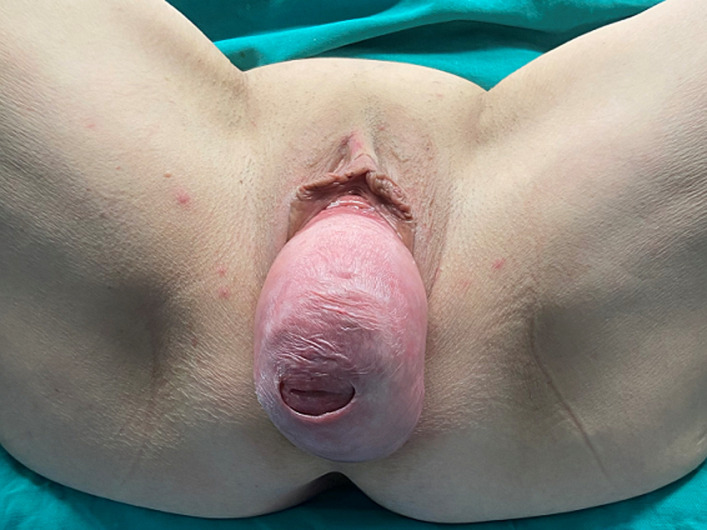
total uterine prolapse associated with bilateral hydronephrosis and deterioration of renal function

**Clinical findings:** the patient reported a feeling of heaviness in the vagina and vulva for about five years. The patient reported no urinary incontinence, but frequent urination and episodes of dysuria rarely associated with urinary retention. During the gynecological examination, total uterine prolapse (fourth stage) with eversion of the vaginal wall was found, without extensive ulcers along the vaginal mucosa and perineum (Figure 1). Cytological examination of cells taken from the ectocervix and endocervix was negative for malignancy.

**Figure 2:**
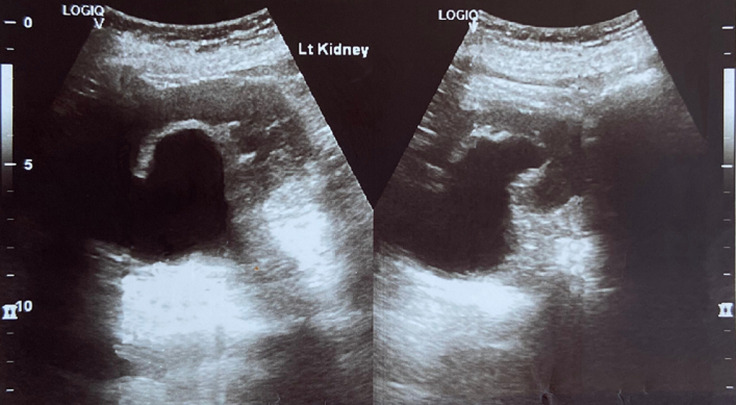
preoperative ultrasound imaging of the right kidney

**Figure 3:**
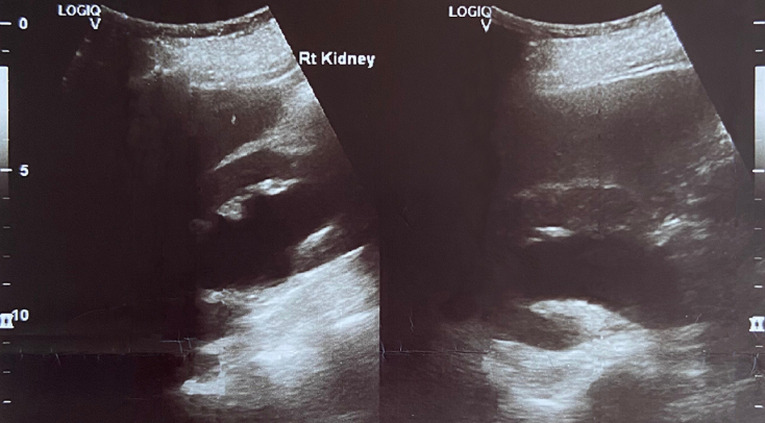
preoperative ultrasound imaging of the left kidney

**Figure 4:**
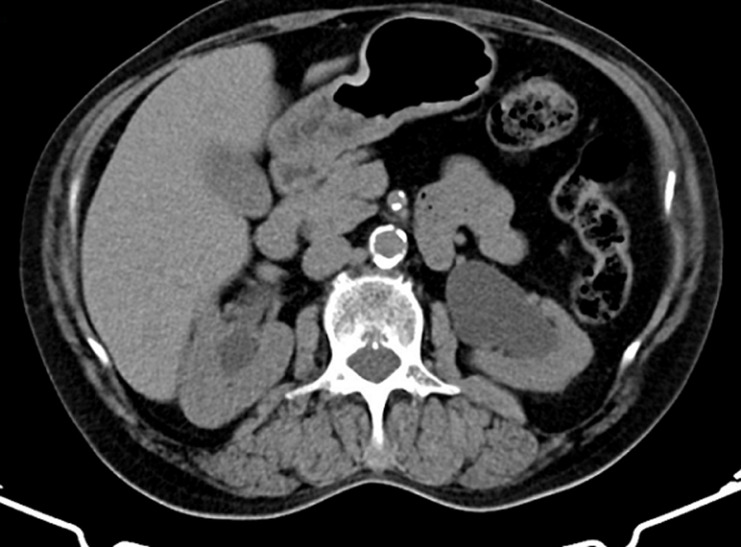
preoperative CT imaging of the abdomen

**Diagnostic assessment:** transvaginal ultrasound revealed a uterus of regular size for the patient's age, without space-occupying lesions. The endometrium was atrophic. Imaging of the ovaries was also without pathological findings. The renal ultrasound showed hydronephrosis bilaterally with severe dilatation of the ureters in their initial part and thinning of the renal cortical thickness (Figure 2, Figure 3). An abdominal computed tomography scan was recommended by the nephrologists to investigate renal dysfunction, confirming the ultrasound findings. Apart from the total uterine prolapse, no other coexisting morbidity was depicted that could be associated with the impairment of renal function (Figure 4). On the admission of the patient to our clinic, from the laboratory analysis, the findings: Ht 37.3%, Hb 12.4gr/dl, PLT 215x10^3^/ml, Urea 85mg/dl, Creatinine 2.2mg/dl, Na 138mEq/lt, K 4.1mEq/lt. The biochemical parameters were within normal ranges. Inflammatory markers were negative. Urine culture was without indication of urinary tract infection.

**Therapeutic intervention:** bilateral hydrouretero nephrosis was considered the main cause of the renal dysfunction as a consequence of the total uterine prolapse and surgical treatment for the pelvic floor damage was decided. After a detailed consultation with the patient and her relatives about the therapeutic approach to the problem, a vaginal hysterectomy was performed with simultaneous correction of the weakening of the anterior and posterior vaginal walls. No immediate complications related to the surgery were reported.

**Figure 5:**
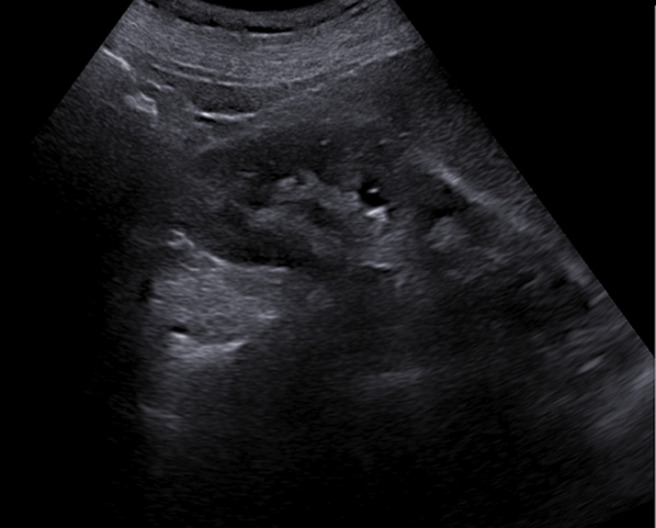
postoperative ultrasound imaging of the right kidney

**Figure 6:**
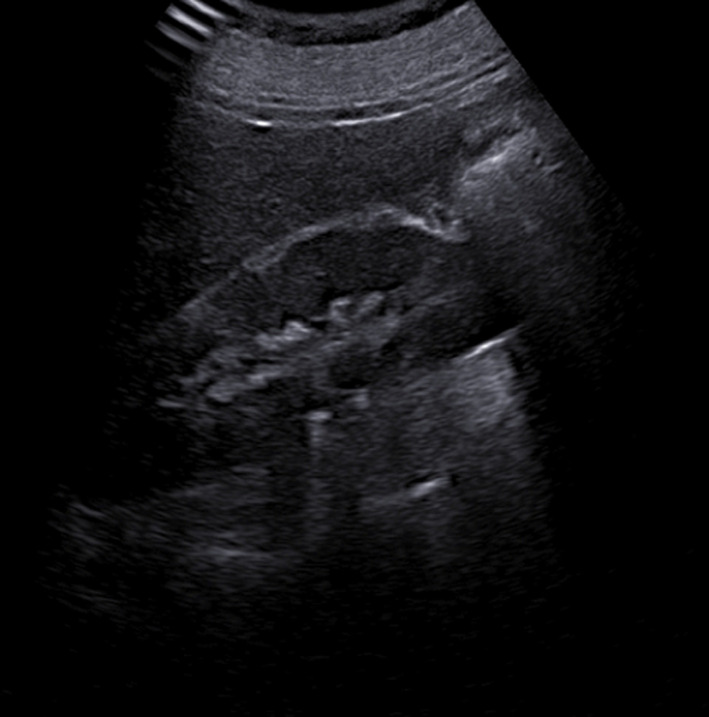
postoperative ultrasound imaging of the left kidney

**Follow-up and outcomes:** after a smooth postoperative course and immediate undisputed improvement of renal function (serum creatinine 1.7 mg/dl), the patient was discharged from our clinic on the fifth day of hospitalization. Three months later, a complete restoration of renal morphology and function was found. The kidneys were visualized with normal echogenicity, normal perfusion and without dilatation of the pelvicalyceal system (Figure 5, Figure 6). Serum creatinine was 1.1 mg/dl. To this day, the patient remains under regular follow-up at the Nephrology and Gynecology outpatient clinic of our hospital.

**Informed consent:** this was sought and obtained from the patient. Anonymity was maintained for confidentiality.

## Discussion

Pelvic organ prolapse is often associated with mild hydronephrosis. Severe bilateral hydroureteronephrosis with renal dysfunction, as in our case, is rarely associated with pelvic organ prolapse [6]. Renal dysfunction ranging from acute to chronic renal failure in most neglected cases can result in end-stage renal disease [7]. The exact pathogenetic mechanism has not been completely clarified. Various theories have been proposed that can´t explain the mechanism of hydronephrosis. Many authors suggest that the extravesical segment of the ureters pulled downward by the prolapsed uterus may cause entrapment of the ureters against the fundus of the uterus and lead to severe obstructive uropathy and renal impairment. This theory, although it could explain many cases of chronic obstructive renal disease due to neglected uterine prolapse, nevertheless cannot explain the unilateral development of the disease, nor its presence in women with vaginal vault prolapse after hysterectomy. In these patients, the weakened supporting ligaments are considered to be able to compress the ureters, due to the descent of the vaginal vault towards the vagina opening [8,9].

The coexistence of lower urinary tract symptoms and diseases is common in patients with uterine prolapse. The change in the shape and anatomical position of the bladder due to the prolapsed uterus and cervix can cause an increase in the post-void residual urine volumes and the development of dysuria. Frequent urination, urge to urinate, bladder overfilling from urinary retention, urinary retention, recurrent urinary tract infections, and in addition, feeling of pelvic pressure, vaginal swelling, bowel dysfunction, and/or sexual dysfunction are symptoms believed to be related to pelvic organ prolapse and may have a significant impact on the quality of life for these patients [10]. In patients with advanced-grade of uterine prolapse, hydroureteronephrosis, which was first described by Brettauer and Rubin in 1923, is a severe complication [11]. This complication, which can easily be overlooked by medical professionals, if not treated promptly and effectively, can significantly affect renal function and cause irreversible damage to the kidneys [6]. In our patient, bilateral hydroureteronephrosis associated with total uterine prolapse due to obstructive uropathy caused an increase in serum creatinine and deterioration of renal function, prompt treatment restored renal function to normal levels. Even in complicated cases with coexisting etiological factors for renal disease, the presence of a severe grade of uterine prolapse and renal dysfunction should urge the clinician to rule out uterine prolapse-related renal impairment.

Diagnosis of obstructive renal disease in patients with pelvic organ prolapse cannot be based on clinical findings. Modern imaging methods for the urinary system have a decisive role in assessing the morphological function of kidneys. Renal ultrasound, abdominal computed tomography, and magnetic resonance imaging are able to estimate with greater accuracy the extent of damage at the level of the kidneys and ureters and contribute to the best planning of remedial operations on the pelvic floor. Renal ultrasound is a first-line examination, with which it is easily possible to establish a significant swelling of the kidneys and a significant grade of bilateral dilatation of the pelvicalyceal system in patients with pelvic organ prolapse [12]. Abdominal computed tomography confirms the findings of renal ultrasound and is also able to exclude other causes of renal dysfunction, thus attributing the varying grade of renal failure to pelvic organ prolapse [3]. In our patient, the preoperative ultrasound imaging of kidneys in combination with abdominal computed tomography scan established the diagnosis of obstructive uropathy related to total uterine prolapse, a well-timed treatment that contributed effectively to the normalization of renal function.

In cases of moderate and severe pelvic organ prolapse, surgery seems to be the main choice of treatment. Conservative treatment with vaginal pessary placement is difficult and does not yield the expected result [13]. Especially in severely neglected cases of pelvic organ prolapse with the presence of mucosal ulcers, vaginal pessary placement should be contraindicated [14]. The most appropriate surgical approach in cases of complete uterine prolapse with coexisting hydroureteronephrosis and deteriorating impairment of renal function seems to be the vaginal approach. Vaginal hysterectomy with simultaneous repair of the cystocele and rectocele and placement of prosthetic meshes supporting the pelvic floor in the majority of cases is expected to ensure the best postoperative support of the vaginal vault and complete restoration of renal morphology and function [15,16]. Similarly, in our patient, after performing a vaginal hysterectomy with simultaneous repair of the cystocele and rectocele, re-examination as part of regular follow-up three months later, morphology and functionality of the kidneys were fully restored. Also, recent data suggest that transvaginal suspension of the high uterosacral ligament is estimated to provide good long-term anatomical results with the excellent support of the vaginal vault, being an alternative reconstructive surgery for the majority of moderate to severe cases of pelvic organ prolapse [17,18].

## Conclusion

Chronic obstructive uropathy due to pelvic organ prolapse, although uncommon, can nevertheless cause severe renal disease. Severe hydronephrosis can cause permanent renal parenchymal damage and lead to renal failure with a significantly increased risk of morbidity and mortality. Thus, on-time and correct diagnosis for the therapeutic approach of similar cases and/or complicated cases with the coexistence of etiological factors for renal disease nowadays must be the main concern of the modern obstetrician-gynecologist, in order to ensure the best health and quality of life of these patients.

## References

[1] Haylen BT, Maher CF, Barber MD, Camargo S, Dandolu V, Digesu A (2016). An International Urogynecological Association (IUGA) / International Continence Society (ICS) Joint Report on the Terminology for Female Pelvic Organ Prolapse (POP). Neurourol Urodyn.

[2] Hall AF, Theofrastous JP, Cundiff GW, Harris RL, Hamilton LF, Swift SE (1996). Interobserver and intraobserver reliability of the proposed International Continence Society, Society of Gynecologic Surgeons, and American Urogynecologic Society pelvic organ prolapse classification system. Am J Obstet Gynecol.

[3] Lucassen EA, la Chapelle CF, Krouwel E, Groeneveld M (2019). Renal failure caused by severe pelvic organ prolapse. BMJ Case Rep.

[4] Dancz CE, Walker D, Thomas D, Özel B (2015). Prevalence of Hydronephrosis in Women With Advanced Pelvic Organ Prolapse. Urology.

[5] Siddique M, Ingraham C, Kudish B, Iglesia CB, Polland A (2020). Hydronephrosis Associated With Pelvic Organ Prolapse: A Systematic Review. Female Pelvic Med Reconstr Surg.

[6] Razzak L, Saulat S (2021). Renal dysfunction due to advance pelvic organ prolapse. J Pak Med Assoc.

[7] Dongol A, Joshi KS, KC S (2013). Renal impairment among patients with pelvic organ prolapse in a tertiary care center. Kathmandu Univ Med J (KUMJ).

[8] Lieberthal F, Frankenthal Junior L (1941). The mechanism of ureteral obstruction in prolapse of the uterus. Surg Gynaecol Obstet.

[9] Farthmann J, Watermann D, Zamperoni H, Wolf C, Fink T, Gabriel B (2017). Pelvic organ prolapse surgery in elderly patients. Arch Gynecol Obstet.

[10] Raju R, Linder BJ (2021). Evaluation and Management of Pelvic Organ Prolapse. Mayo Clin Proc.

[11] Brettauer J, Rubin IC (1923). Hydroureter and hydronephrosis: a frequent secondary finding in cases of prolapse of the uterus and bladder. American Journal of Obstetrics and Gynecology.

[12] Kurt S, Guler T, Canda MT, Demirtas Ö Tasyurt A (2014). Treatment of uterine prolapse with bilateral hydronephrosis in a young nulliparous woman; a new minimally invasive extraperitoneal technique. Eur Rev Med Pharmacol Sci.

[13] Collins S, Lewicky-Gaupp C (2022). Pelvic Organ Prolapse. Gastroenterol Clin North Am.

[14] Leanza V, Di Stefano A, Paladino EC, Rivoli L, Distefano REC, Palumbo M (2022). Stasis ulcer and hydronephrosis after severe genital prolapse: a case report. J Med Case Rep.

[15] Ellington DR, Richter HE (2013). Indications, contraindications, and complications of mesh in surgical treatment of pelvic organ prolapse. Clin Obstet Gynecol.

[16] Leanza V, Ciotta L, Vecchio R, Zanghì G, Maiorana A, Leanza G (2015). Hydronephrosis and utero-vaginal prolapse in postmenopausal women: management and treatment. G Chir.

[17] Zhang YH, Lu YX, Liu X, Liu JX, Shen WJ, Zhao Y (2019). A five-year analysis of effect on transvaginal high uterosacral ligament suspension with or without native-tissue repair for middle compartment defect. Zhonghua Fu Chan Ke Za Zhi.

[18] Shen WJ, Lu YX, Liu X, Liu JX, Duan L, Zhang YH (2019). Effectiveness of vaginal high uterosacral ligament suspension for treatment of recurrent pelvic organ prolapse. Zhonghua Fu Chan Ke Za Zhi.

